# Impact of in ovo myo-inositol administration on immune modulation in broiler chickens

**DOI:** 10.3389/fimmu.2026.1843984

**Published:** 2026-05-21

**Authors:** Tanja Hofmann, Nataliia Shomina, Vera Sommerfeld, Markus Rodehutscord, Korinna Huber, Volker Stefanski

**Affiliations:** Institute of Animal Science, Faculty of Agricultural Sciences, University of Hohenheim, Stuttgart, Germany

**Keywords:** broiler chickens, immune cells, immune system, in ovo injection, myo-inositol

## Abstract

Myo-inositol (MI) plays an important role in intracellular signaling, cell proliferation, and cell growth, and has been associated with positive effects on the immune system and health in fish. However, its potential immunological effects in poultry have not been investigated yet. This study aimed to evaluate the effects of in ovo MI administration on immune parameters in broiler chickens after hatching. A total of 480 fertilized Ross 308 eggs were randomly assigned as hatched to four groups and injected on embryonic day 17 with either 12 µmol/mL MI (MI-12), 24 µmol/mL MI (MI-24), 0.9% saline (positive control, PC), or left untreated (negative control, NC). After hatching, broilers were housed in floor pens. On day 35, eight birds per treatment group were sampled for analysis of leukocyte subsets and antibody concentrations in blood. Overall, in ovo MI administration had only slight effects on the immune parameters. Birds in the MI-24 group had lower plasma IgM concentrations and showed a tendency toward lower CD4^+^ cell numbers, while no effects were observed on the numbers of other investigated immune cell populations or antibody classes. The current findings should be considered hypothesis-generating given an unbalanced sex distribution across groups. Together, the results suggest that in ovo MI administration at the end of embryogenesis may influence immune cell function rather than cell development. Moreover, neither piercing the eggshell nor injecting fluid affected the immune parameters analyzed, indicating that this technique does not compromise the immune system at this stage of embryonic development.

## 1 Introduction

Myo-inositol (MI) has emerged as a promising feed additive in animal nutrition due to its involvement in various physiological processes such as intracellular signaling, cell proliferation, cell cycle, apoptosis, metabolism, oxidative stress, and cell growth (1–3). Myo-inositol is the most abundant and biologically active isomer of inositol (cyclohexane-1,2,3,4,5,6-hexol) in nature and can be acquired through endogenous biosynthesis from glucose-6-phosphate, exogenous food intake, and the breakdown of phosphatidylinositol, phosphoinositides, and inositol phosphates (3, 4).

Myo-inositol is crucial for reproduction, physiology and health in both humans and animals (5–10). In fish, MI positively modulates several immunological responses, including the regulation of inflammatory cytokines, reduction of apoptosis, enhancement of lysozyme and phagocytic activity, and stimulation of immune cell proliferation (11–17), suggesting that MI could enhance non-specific and specific immune responses. In chickens, dietary MI supplementation has been shown to modulate performance and metabolic processes (3, 18–21); however, its effects on the immune system have not yet been investigated. Current information on the effects of MI on the immune system of poultry is derived from studies involving dietary phytase supplementation, which indirectly increases MI availability (22, 23). Sommerfeld et al. (23) demonstrated that a phosphorus-reduced diet resulted in altered MI levels in the gastrointestinal tract, blood, and eggs of brown and white hens, a difference that coincided with strain-specific immune responses (24). However, this indirect approach does not allow to isolate the specific effects of MI, as the observed outcomes may result from a combination of increased MI release, reduced levels of anti-nutritional inositol phosphates, and improved phosphorus availability.

As precocial birds, chicks have already reached an advanced stage of structural and functional development at the time of hatching (25). Most of the immune system’s development is completed by the late embryonic phase in chickens, but maturation and response of the immune system to antigens increase with age after hatching (26–29). Consequently, the prenatal phase is of utmost importance for shaping offspring phenotypes with potential long-lasting consequences for later life (30). In contrast to mammalian embryos, which remain in continuous exchange with the mother throughout gestation, avian embryos must rely solely on a fixed reserve of nutrients deposited in the egg prior to incubation for their development (31). Hence, the success of embryonic development, including the immune system, depends on the adequate presence of essential nutrients in the fertile egg (32). In ovo administration of nutrients (33–35) and other bioactive substances such as probiotics and synbiotics (36–38), as well as plant extracts (39) have been proven to accelerate the development and maturation of the immune system and to improve cellular and humoral immune response of chickens. In line with these findings, we recently reported the effects of in ovo administration of MI on growth performance and metabolic profiles in broiler chickens (40).

The aim of the present study was to extend this knowledge by examining the impact of in ovo MI administration on the immune system and evaluating its potential to influence immune development during late embryogenesis. We hypothesized that in ovo administration of MI would (1) increase antibody levels based on the role of MI in enhancing the overall function of immune cells, and (2) increase the absolute numbers of immune cells based on the role of MI in regulating cell proliferation and apoptosis.

## 2 Materials and methods

### 2.1 Experimental design

The study was conducted at the Agricultural Experimental Station of the University of Hohenheim, Germany, as a joint project to the DFG Research Unit P-Fowl – Inositol phosphates and myo-inositol in the domestic fowl: Exploring the interface of genetics, physiology, microbiome, and nutrition (https://p-fowl.uni-hohenheim.de/). Blood samples for the immunological analyses reported here were collected in a study focusing on in ovo effects of MI (40). All procedures were approved by the local authority’s Animal Ethics Committee (Regional Council Tübingen, approval number HOH 75/24-460a).

In brief, a total of 550 fertilized Ross 308 broiler chicken eggs were obtained from a single 52-week-old breeder flock in Baden-Württemberg, Germany. The eggs were individually weighed and randomly assigned to four experimental groups (n = 120 per group), ensuring a comparable mean egg weight across groups (67.3 ± 0.4 g). The remaining eggs were also incubated to compensate for potential losses due to non-fertilization or early embryonic mortality. All eggs were incubated from embryonic day (ED) 1 to 17 under standard conditions for embryonic development (37.8 °C, 65% relative humidity, and automatic turning every hour). On ED 11, eggs were candled and unfertilized or non-viable eggs were removed. A second candling was conducted on ED 16 to confirm ongoing embryo viability.

In ovo administration of MI was conducted on ED 17 in viable embryos. Prior to injection, eggshells were disinfected with 70% ethanol. A small hole was pierced into the blunt end of each egg using a 19-gauge needle. Subsequently, 1.0 mL of the respective treatment solution was injected into the amniotic cavity using a sterile 23-gauge needle. After injection, the shell opening was sealed with hot paraffin wax to prevent contamination and moisture loss. The experimental groups included: (1) MI 0.012 M group (MI-12) – injected with 1.0 mL of a 12 µmol/mL MI solution, (2) MI 0.024 M group – injected with 1.0 mL of a 24 µmol/mL MI solution (MI-24), (3) positive control group (PC) – injected with 1.0 mL of sterile 0.9% saline solution, and (4) left untreated, negative control group (NC), serving as a baseline for natural embryonic development. The concentrations of MI used for in ovo injections were selected based on natural MI concentrations of at least 12 µmol in egg albumen (23). Myo-inositol (cell culture grade; # J62886.18, Thermo Fisher GmbH, Kandel, Germany) was dissolved in 0.9% saline (# 13423, Honeywell Fluka™, Germany), and all solutions were sterilized by autoclaving.

Egg handling time outside the incubator did not exceed 20 minutes, and eggs of the NC group were also removed to control for handling effects. Following in ovo treatment, all eggs were transferred to a hatcher under standard conditions until hatching.

On the day of hatching, a total of 384 unsexed chicks (96 per experimental group) were allocated to 32 floor pens (110 × 230 cm ground area, 200 cm height) on dedusted wood shavings, with 12 chicks housed per pen. Each treatment group was assigned to separate pens, with a total of eight replicate pens per group. Pens were arranged in a randomized complete block design to minimize potential location effects in the barn. To ensure uniformity, chicks were weighed per pen, and pen composition was adjusted to achieve an equal average body weight at placement across all pens within each treatment group. All birds were housed under equal environmental and husbandry conditions and received a commercial starter diet (ME – 12.4 MJ/kg; CP – 21.5%; Landkornstarter, Deuka) from day 0 to 14, followed by a grower diet (ME – 12.4 MJ/kg; CP – 20%; Landkornmast, Deuka) from day 14 to 35 for ad libitum consumption.

### 2.2 Blood sampling

At 35 days of age, the broiler closest to the mean weight of each pen was slaughtered by stunning, using a gas mixture of 35% CO_2_, 35% N_2_, and 30% O_2_ followed by decapitation (n = 8 per treatment group). Since sex could not be determined prior to slaughter, a balanced sex distribution among treatment groups could not be ensured in advance. Trunk blood was collected in 5 mg/mL EDTA tubes (Sigma Aldrich, St. Louis, MO, USA). For flow cytometric analysis, the blood was fixed by TransFix^®^ reagent (Caltag Medsystems Ltd., UK) in accordance with the manufacturer’s instructions. Unfixed blood samples for antibody analyses were centrifuged (15 min at 2000 × *g* and 4 °C) and subsequently stored at −20 °C until measurement.

### 2.3 Flow cytometric analysis for the identification of leukocyte subsets

Flow cytometric analyses for identifying and quantifying leukocyte subsets in whole blood were performed using the no-lyse, no-wash method as described by Seliger et al. (41). Samples were stained with fluorescently conjugated antibodies and gated based on the strategy outlined by Hofmann et al. (42). Chicken-specific fluorochrome-conjugated antibodies targeting distinct cell surface markers were employed as follows: anti-CD45-APC (clone LT40, Cat# 8270-11, RRID: AB_2796475), anti-Monocyte/Macrophage-PE (clone Kul01, Cat# 8420-09, RRID: AB_2796566), anti-CD4-Pacblu (clone CT-4, Cat# 8210-26, RRID: AB_2796434), anti-CD8α-FITC (clone CT-8, Cat# 8220-02, RRID: AB_2796439), anti-Bu-1-FITC (clone AV20, Cat# 8395-02, RRID: AB_2796542) (all SouthernBiotech, Birmingham, AL, USA), anti-CD41/61-PE (clone 11C3, Cat# MCA2240GA, RRID: AB_3100681; BioRad, Santa Rosa, CA, USA), and anti-TCRγδ-PerCP (clone TCR1, Cat# NBP1-28275PCP, RRID: AB_3204033; Novus Biologicals, Centennial, CO, USA). Flow cytometric analyses were conducted using a BD FACSCanto™ II (BD Biosciences, Heidelberg, Germany) equipped with 488 nm (blue), 630 nm (red), and 405 nm (violet) lasers, and BD FACSDiva™ Software. Single-stained antibody controls were included for fluorescence compensation to correct for spectral overlap between fluorochromes and to ensure accurate interpretation of multicolor flow cytometry data. In addition, fluorescence minus one (FMO) control were used to verify proper gating and to distinguish true positive signals from background fluorescence. For each sample, a mouse IgM isotype control conjugated to APC (clone 11E1, Cat# 0101-11, RRID: AB_2793838; SouthernBiotech) and an unstained control were employed to assess nonspecific antibody binding and baseline autofluorescence. Data were gated based on specific combinations of surface marker expression (cluster of differentiation, CD) following exclusion of cell doublets: thrombocytes (CD45dim/CD41/61+), monocytes (CD45+/Kul01+), CD4+ T cells (CD45+/CD4+/TCRγδ−), CD8α+ T cells (CD45+/CD4−/TCRγδ−/CD8α+), γδ T cells (CD45+/Kul01−/CD4−/TCRγδ+), and B cells (CD45+/Kul01−/Bu-1+). Heterophils in blood were identified based on their forward and side scatter characteristics. Absolute leukocyte numbers were determined using BD Trucount™ tubes (BD Biosciences) according to the manufacturer’s instructions. Total T cells were defined as the combined number of CD4+ cells, CD8α+ cells, and γδ T cells. Lymphocyte counts were derived from the cumulative sum of T cells and B cells.

### 2.4 Enzyme-linked immunosorbent assay for antibody quantification

Antibody concentration was determined by a Sandwich enzyme-linked immunosorbent assay (ELISA) as described in detail previously (43). For plate coating, goat anti-chicken IgY Fc (100 ng/well), goat anti-chicken IgM (200 ng/well), and goat anti-chicken IgA (200 ng/well) were used (all Bethyl Laboratories, Montgomery, TX, USA). For detection, horseradish peroxidase-conjugated goat anti-chicken IgY Fc (diluted 1:100,000), goat anti-chicken IgM (diluted 1:200,000), and goat anti-chicken IgA (diluted 1:200,000) were applied (all Bethyl Laboratories). Prior to analysis, plasma samples were diluted 1:200,000 for IgY, 1: 2,000 for IgM and 1: 1,500 for IgA. Optical density was measured at 450 nm using a Spark multimode microplate reader (Tecan, Switzerland). Antibody concentrations were calculated relative to the absorbance of the standard curves that were generated from a pooled plasma sample, in which IgY, IgM, and IgA concentrations had been previously quantified using chicken-specific ELISA kits (all Bethyl Laboratories).

The intra-assay coefficient of variation (CV), calculated from five duplicate measurements within a single ELISA plate, was 8.8% for IgY, 5.5% for IgM, and 4.6% for IgA. The inter-assay CV, determined across independent assay runs using the same pooled plasma control, was 3.9% for IgY, 4.4% for IgM, and 8.9% for IgA.

### 2.5 Statistical analysis

Statistical analyses were conducted using SAS software (Version 9.4; SAS Institute Inc., Cary, NC). A linear mixed model was applied using the PROC MIXED procedure, after testing residuals for normal distribution and homogeneous error variance through graphical check of residual plots (44). If model assumptions were not fulfilled, logarithmic transformations were used to stabilize variance and meet the distribution assumption. In this case, results were back-transformed for presentation. Degrees of freedom were estimated by the method of Kenward-Roger, and variance components were estimated using the restricted maximum likelihood (REML) method. The model included treatment and sex as fixed effects and block as a random effect. Additionally, MI-injected groups (MI-12 + MI-24) and no-MI groups (PC + NC) were compared. To account for potential heteroscedasticity, a repeated statement was included with treatment as the grouping factor. The results are presented as LSmeans with their SEM. Differences were considered statistically significant at P < 0.05, and trends were accepted at 0.05 ≤ P < 0.10.

## 3 Results

### 3.1 Antibody concentration

**Figure 1:**
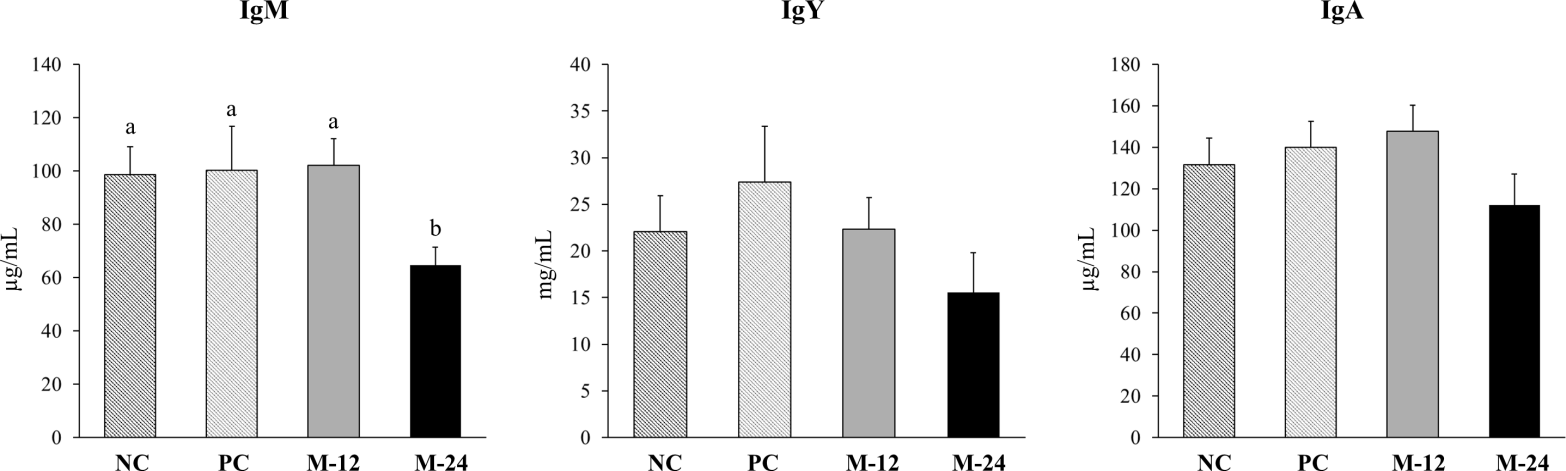
Antibody concentrations in the plasma of 35-day-old broilers that received in ovo treatment on ED 17 with either 0.012 M myo-inositol (MI-12; n = 8), 0.024 M myo-inositol (MI-24; n = 8), saline (PC; n = 8), or left untreated (NC; n = 8). Different lowercase letters indicate statistically significant differences between treatment groups at P < 0.05.

Mixed linear model analysis revealed a significant treatment effect of in ovo MI administration on IgM levels (P = 0.026), with birds from the MI-24 group having lower IgM concentrations compared to all other groups (**Figure 1**), while there was no effect for IgY (P = 0.425) and IgA (P = 0.314). No significant effect of sex on plasma antibody concentrations was observed (P > 0.05) (**Supplementary Table 1**). Moreover, no difference between MI-injected groups and no-MI groups was observed (P > 0.05).

### 3.2 Number and relative proportion of leukocyte subsets

Mixed linear model analysis revealed no effect of treatment (P > 0.05) on either the absolute numbers (**Table 1**) or the relative proportions (**Supplementary Table 2**) of leukocyte subsets in the blood of 35-day-old broiler chickens treated in ovo with MI on ED 17, except a statistical trend in the number of CD4^+^ cells (P = 0.069). CD4^+^ cell numbers were highest in the PC group and lowest in the MI-24 group. No differences between MI-injected groups and no-MI groups were observed (P > 0.05).

**Table 1:** Absolute numbers of total leukocytes and their subsets per µL blood, as well as the heterophil-to-lymphocyte (H/L) ratio and the CD4^+^/CD8^+^ cell ratio in 35-day old broilers treated in ovo on embryonic day 17 with either 0.012 M myo-inositol (MI-12; n = 8), 0.024 M myo-inositol (MI-24; n = 8), saline (PC; n = 8), or left untreated (NC; n = 8).

Parameter	NC	PC	MI-12	MI-24	P-value	
					Treatment	MI vs no-MI^§^
Leukocytes	67,333 ± 5,373	58,223 ± 2,626	65,887 ± 3,662	61,487 ± 3,576	0.300	0.837
Heterophils	52,145 ± 4,821	41,445 ± 3,132	51,031 ± 4,063	45,763 ± 3,802	0.192	0.677
Monocytes	292 ± 97	211 ± 70	217 ± 72	384 ± 147	0.488	0.743
CD4^+^ cells	5,536 ± 424	6,180 ± 354	5,612 ± 504	4,950 ± 547	0.069	0.475
CD8^+^ cells	2,423 ± 174	2,631 ± 206	2,275 ± 217	2,915 ± 364	0.468	0.776
γδ T cells	2,573 ± 229	2,819 ± 289	2,435 ± 250	2,283 ± 338	0.531	0.213
B cells	2,458 ± 326	2,599 ± 322	2,056 ± 322	3,063 ± 373	0.260	0.654
Thrombocytes	44,025 ± 2,830	45,868 ± 2,919	43,935 ± 2,796	41,237 ± 3,027	0.656	0.517
CD4^+^/CD8^+^	2.30 ± 0.15	2.41 ± 0.26	2.54 ± 0.24	1.88 ± 0.28	0.431	0.917
H/L	3.89 ± 0.35	2.84 ± 0.35	4.04 ± 0.53	3.40 ± 0.47	0.213	0.320

^§^ MI treatment (MI-12 + MI-24) vs. controls (PC + NC).

Moreover, mixed linear model analysis revealed a significant effect of sex. Males had higher relative proportions of CD8^+^ cells (P = 0.044) than females (**Table 2**), which is reflected in a lower CD4^+^/CD8^+^ ratio (P = 0.028) (**Table 3**). A tendency was also found for lower numbers of CD4^+^ cells (P = 0.079) and higher numbers of CD8^+^ cells (P = 0.097) per mL blood in males compared to females (**Table 3**).

**Table 2:** Relative proportion of leukocyte subsets in blood of female (n = 19) and male (n = 13) 35-day old broiler chickens.

Parameter	Female	Male	P-value
Heterophils	75.51 ± 1.97	73.25 ± 2.31	0.475
Monocytes	0.35 ± 0.10	0.52 ± 0.18	0.434
CD4^+^ cells	9.37 ± 0.70	8.45 ± 0.77	0.407
CD8^+^ cells	3.45 ± 0.28^b^	4.54 ± 0.45^a^	0.044
γδ T cells	3.85 ± 0.35	4.25 ± 0.46	0.451
B cells	3.50 ± 0.36	4.14 ± 0.53	0.338

Different superscript letters indicate statistically significant differences between treatment groups at P < 0.05.

**Table 3:** Absolute numbers of total leukocytes and their subsets, as well as the heterophil-to-lymphocyte (H/L) ratio and the CD4^+^/CD8^+^ cell ratio in the blood of female (n = 19) and male (n = 13) 35-day old broiler chickens.

Parameter	Female	Male	P-value
Leukocytes	66,588 ± 2,908	59,877 ± 3,640	0.204
Heterophils	50,866 ± 3,205	44,326 ± 4,009	0.256
Monocytes	233 ± 64	307 ± 98	0.483
CD4^+^ cells	6,152 ± 401	5,011 ± 403	0.079
CD8^+^ cells	2,327 ± 163	2,795 ± 200	0.097
γδ T cells	2,536 ± 238	2,504 ± 270	0.920
B cells	2,436 ± 222	2,652 ± 275	0.571
Thrombocytes	41,684 ± 2,224	45,885 ± 2,848	0.201
CD4^+^/CD8^+^	2.60 ± 0.18^a^	1.98 ± 0.17^b^	0.028
H/L	3.76 ± 0.37	3.28 ± 0.40	0.436

Different superscript letters indicate statistically significant differences between treatment groups at P < 0.05.

## 4 Discussion

The findings indicate that in ovo administration of MI can influence certain immune parameters in broiler chickens; however, the effects were limited and did not support the initial hypothesis of increased antibody levels and immune cell abundance. In contrast, in ovo administration of high MI concentration resulted in reduced plasma IgM concentrations in 35-day-old broiler chickens. This suggests that early B-cell activation, and consequently the primary antibody response, were affected. The phosphoinositide 3-kinase (PI3K) pathway, which regulates cell survival, proliferation, metabolism, and resistance to oxidative stress (45) may represent a potential contributing mechanism, as MI serves as a precursor for signaling molecules that activate the PI3K pathway. Mature naïve B cells depend on balanced PI3K signaling for proper development and IgM expression (46). High in ovo MI concentrations may have altered PI3K pathway activity, resulting in less favorable conditions for IgM production. However, MI administration on ED 17 did not alter IgY and IgA concentrations, thus indicating that immunological processes associated with the secondary antibody response were not influenced. Moreover, the numbers of all circulating immune cell subsets were unaffected, except for a trend toward lower CD4^+^ cell numbers. These findings suggest that in ovo MI administration may influence immune cell function rather than immune cell development in 35-day-old broiler chickens. From a broader perspective, the overall effect of in ovo MI administration at the end of embryogenesis on the immune system in chickens appears limited. Nevertheless, as immune parameters were assessed only at day 35 post hatch, transient effects during early post-hatch development cannot be excluded. Importantly, however, the NC group did not differ from the other treatment groups, indicating that neither eggshell piercing nor fluid injection adversely affected the assessed immune parameters.

This first study on effects of in ovo MI application on the immune system was conducted within a project (40) with a predefined experimental design primarily focused on growth performance and metabolic profiles, including a fixed timing of in ovo administration that could not be modified for the present investigation. This constraint should be considered when drawing conclusions regarding the immune system. Myo-inositol was applied at ED 17, a time point typically used for the delivery of substances into the amniotic fluid for in ovo feeding (47). From ED 17 to hatching, the amniotic fluid is swallowed by the embryo, allowing for the absorption of nutrients that support the embryo’s survival and health immediately after hatching (48–50). While immune maturation and the capacity to respond to antigens continue to increase post-hatching, most of the immune system’s development is already completed by the late embryonic phase. The thymus and the bursa of Fabricius become fully mature by ED 12 and ED 18 respectively, with T and B cells being detectable in the embryo circulation as early as ED 10 – ED 14 (51–53). Therefore, injections at ED 17 may be too late to significantly influence immune cell lineage commitment or expansion. Hence, an earlier administration, aligning with lymphoid organ maturation and lymphocyte differentiation, may be a more optimal time to stimulate the developing embryonic immune system in future studies.

Moreover, the dosage is critically important in in ovo administration, determining beneficial, neutral, or harmful effects (54). The MI concentrations used for in ovo injections in this study were chosen based on the natural MI content of egg albumen (23). In the present experiment, the average MI content of egg albumen measured in non-incubated eggs from the same batch was higher, amounting to 14.5 ± 1.7 µmol (40). Relative to this value, the in ovo injections supplied an approximately 0.8-fold (MI 12) and 1.7-fold (MI 24) increase of the naturally occurring MI content. The high MI dose negatively affected IgM concentration, while no effect was observed in immune cell abundance. This highlights the importance of determining an optimal dose of MI that may positively stimulate the immune system without causing adverse effects.

In addition, plasma and tissue MI concentrations at slaughter were similar across all treatment groups (40), indicating that the administered MI did not cause persisting changes to MI homeostasis. Nevertheless, early exposure may trigger functional or metabolic reprogramming that affects antibody production, while immune cell numbers remain unchanged due to the absence of a sustained proliferative stimulus. This aligns with the determined subtle but consistent metabolic changes, including alterations in energy and protein metabolism, lipid-mediated signaling, and peripheral metabolic pathways (40), which likely reflect a long-lasting reprogramming of cellular function rather than changes in cell abundance.

A limitation to the present study was the unbalanced sex distribution at sampling, as the MI-24 group turned out to comprise exclusively females. Notably, only the MI-24 group exhibited altered antibody concentrations, raising the possibility that the observed effect reflects an interaction between the high MI dose and sex, rather than a treatment effect per se. Although sex was included as a fixed factor in the statistical model, the unbalanced distribution limits the interpretation of potential sex-dependent MI effects, particularly in the MI-24 group. This is especially relevant as slight differences in immune parameters between males and females were observed in this study, consistent with a recent study in chickens (55). Considering this limitation, our findings should be regarded as hypothesis-generating for future studies with larger group sizes to increase statistical power and to ensure balanced sex ratios.

In summary, in ovo administration of 0.024 M MI during late embryogenesis negatively affected IgM antibody concentrations in 35-day-old broiler chickens, indicating that in ovo MI can modulate the developing immune system. High MI concentrations may dysregulate primary antibody production rather than providing beneficial effects. However, before drawing general conclusions, larger studies are needed to systematically evaluate the effects of sex, dosage and timing. Importantly, neither eggshell piercing nor fluid injection adversely affected immune parameters, supporting the use of this technique as a reliable methodological tool.

## Data Availability

The raw data supporting the conclusions of this article will be made available by the authors, without undue reservation.
