# Supplementary material for: Impact of in ovo myo-inositol administration on immune modulation in broiler chickens
Source: Front Immunol. 2026 May 21;17:1843984. doi: 10.3389/fimmu.2026.1843984 (PMC13233394; doi:10.3389/fimmu.2026.1843984)
Supplement: Supplementary file 1 [file DataSheet1.pdf]

Supplementary Material

Supplementary Table 1: Antibody concentration in the plasma of female (n = 19) and male (n = 13) 35-day old broiler chickens.

Parameter	Female	Male	P-value
IgM (μg/mL)	99.43 ± 9.47	81.32 ± 9.49	0.213
IgY (mg/mL)	26.40 ± 3.49	17.29 ± 4.31	0.137
IgA (μg/mL)	141.12 ± 9.17	124.50 ± 11.61	0.322

Supplementary Table 2: Relative proportions of leukocyte subsets among total leukocytes (%) in the blood of 35-day-old broilers that received in ovo treatment on embryonic day 17 with either 0.012 M myo-inositol (MI-12; n = 8), 0.024 M myo-inositol (MI-24; n = 8), saline (PC; n = 8), or left untreated (NC; n = 8).

Parameter	NC	PC	MI-12	MI-24	P-value	
					Treatment	MI vs no-MI§
Heterophils	76.57 ± 1.97	70.41 ± 2.67	76.61 ± 2.55	74.41 ± 2.67	0.282	0.374
Monocytes	0.44 ± 0.13	0.36 ± 0.16	0.33 ± 0.14	0.63 ± 0.24	0.704	0.763
$CD4^+$ cells	8.43 ± 0.71	10.69 ± 0.76	8.63 ± 0.95	8.08 ± 0.85	0.312	0.341
$CD8^+$ cells	3.61 ± 0.23	4.43 ± 0.48	4.53 ± 0.71	3.39 ± 0.41	0.256	0.499
γδ T cells	3.91 ± 0.43	4.88 ± 0.54	3.75 ± 0.41	3.74 ± 0.48	0.178	0.151
B cells	3.59 ± 0.54	4.17 ± 0.62	3.02 ± 0.45	4.65 ± 0.80	0.264	0.522

§ MI treatment (MI-12 + MI-24) vs. controls (NC + PC)
